# Radiation treatment planning and delivery strategies for a pregnant brain tumor patient

**DOI:** 10.1002/acm2.12262

**Published:** 2018-07-30

**Authors:** Zacariah E. Labby, Brendan Barraclough, R. Adam Bayliss, Abigail E. Besemer, David A. P. Dunkerley, Steven P. Howard

**Affiliations:** ^1^ Department of Human Oncology University of Wisconsin – Madison Madison WI USA

**Keywords:** astrocytoma, fetal dose, pregnant patient, radiation therapy

## Abstract

The management of a pregnant patient in radiation oncology is an infrequent event requiring careful consideration by both the physician and physicist. The aim of this manuscript was to highlight treatment planning techniques and detail measurements of fetal dose for a pregnant patient recently requiring treatment for a brain cancer. A 27‐year‐old woman was treated during gestational weeks 19–25 for a resected grade 3 astrocytoma to 50.4 Gy in 28 fractions, followed by an additional 9 Gy boost in five fractions. Four potential plans were developed for the patient: a 6 MV 3D‐conformal treatment plan with enhanced dynamic wedges, a 6 MV step‐and‐shoot (SnS) intensity‐modulated radiation therapy (IMRT) plan, an unflattened 6 MV SnS IMRT plan, and an Accuray TomoTherapy HDA helical IMRT treatment plan. All treatment plans used strategies to reduce peripheral dose. Fetal dose was estimated for each treatment plan using available literature references, and measurements were made using thermoluminescent dosimeters (TLDs) and an ionization chamber with an anthropomorphic phantom. TLD measurements from a full‐course radiation delivery ranged from 1.0 to 1.6 cGy for the 3D‐conformal treatment plan, from 1.0 to 1.5 cGy for the 6 MV SnS IMRT plan, from 0.6 to 1.0 cGy for the unflattened 6 MV SnS IMRT plan, and from 1.9 to 2.6 cGy for the TomoTherapy treatment plan. The unflattened 6 MV SnS IMRT treatment plan was selected for treatment for this particular patient, though the fetal doses from all treatment plans were deemed acceptable. The cumulative dose to the patient's unshielded fetus is estimated to be 1.0 cGy at most. The planning technique and distance between the treatment target and fetus both contributed to this relatively low fetal dose. Relevant treatment planning strategies and treatment delivery considerations are discussed to aid radiation oncologists and medical physicists in the management of pregnant patients.

## 1 INTRODUCTION

Patients requiring radiation therapy are seldom simultaneously pregnant. However, when both conditions apply, unique considerations are required from the radiation oncologist and the medical physicist. Especially at doses exceeding 10 cGy, the deterministic effects of ionizing radiation on the developing fetus are moderately understood, and linear extrapolations of stochastic risk estimates are commonplace.^1^, ^2^ Radiation therapy can play a net‐beneficial role in the management of a pregnant patient, but depending on the treatment site, special treatment planning techniques to reduce peripheral dose and/or fetal radiation shields may be necessary.

While breast cancer and hematologic malignancies make up the preponderance of cancers seen in a pregnant population, other tumor types are found with some frequency, including brain tumors.^3^ Common brain malignancies (e.g., gliomas) found in a patient population of child‐bearing age are often treated with shaped radiation fields or arcs that enter the patient's head from many angles, including so‐called “vertex” beams. It may be possible to achieve a clinically acceptable plan while substantially reducing peripheral dose by modifying these standard treatment planning strategies. Numerous reports have detailed planning strategies to reduce peripheral dose.^4^, ^5^, ^6^, ^7^, ^8^, ^9^, ^10^ While IMRT is a common choice for intracranial treatments, IMRT often results in higher peripheral dose than 2D‐ or 3D‐conformal treatment techniques.^5^, ^10^

The purpose of this manuscript was to detail the special considerations for a pregnant brain cancer patient recently treated in our clinic, including treatment plan design for both a Varian TrueBeam system and an Accuray TomoTherapy HDA system, peripheral dose estimation and measurement, and other patient management strategies. While prior reports have provided estimates of peripheral dose for various combinations of beam energy and geometry, these reports often apply to a prior generation of treatment delivery system.^1^, ^4^, ^5^, ^6^, ^10^, ^11^, ^12^, ^13^, ^14^ The present manuscript details fetal dose measurements on current‐generation treatment delivery systems and summarizes the anticipated risks to the patient's fetus using published guidance. Finally, we aim to briefly summarize some of the relevant literature on fetal dose in radiation therapy.

## 2 METHODS AND MATERIALS

### 2.A Patient details

**Figure 1:**
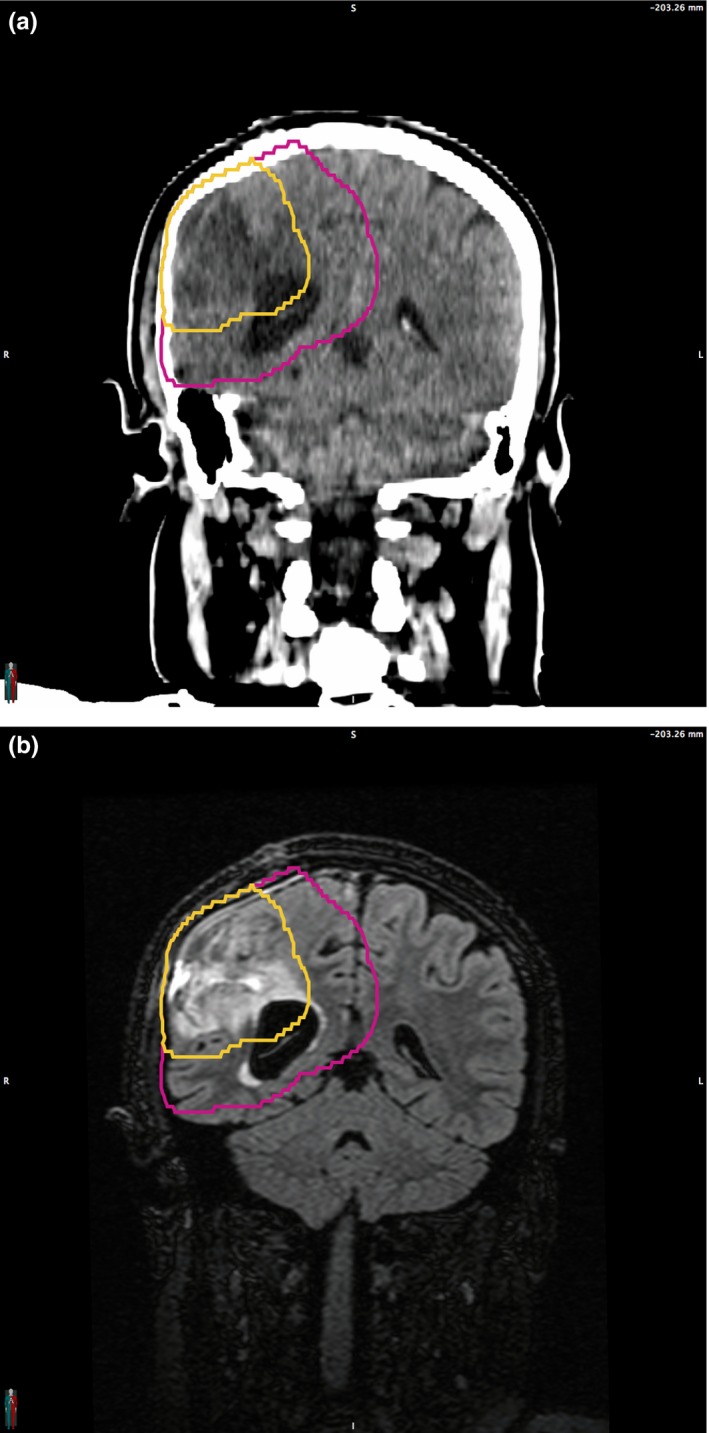
Coronal images of the patient's planning images; (a) simulation CT scan and (b) registered T2 FLAIR MR scan. The primary and boost target volumes are shown in magenta and gold, respectively.

The patient detailed in this report is a 27‐year‐old pregnant female. She was simulated for treatment to her grade 3 astrocytoma resection cavity during gestational week 17. The patient was prescribed 50.4 Gy in 28 fractions to a primary target volume to be followed sequentially by a 9 Gy boost in five fractions to a smaller volume. Medical images with delineated target volumes are shown in Fig. 1. Standard departmental brain planning constraints were ordered for this patient, including D(0.03 mL) < 54 Gy for the brainstem, optic chiasm, and optic nerves; mean dose < 35 Gy and D(0.03 mL) < 40 Gy for the cochleae; D(0.03 mL) < 7 Gy for the lenses of the eyes; and D(0.03 mL) < 45 Gy for the spinal cord. At least 95% of the target volume was covered with 99% of the prescription dose in each treatment plan, and target hotspots were maintained less than 110%.

Fetal dose was assessed at four points of interest bracketing the distances where the fetus could conceivably be located during the patient's course of treatment: (a) the pubic symphysis, (b) the uterine fundus on the date of simulation, (c) the umbilicus, and (d) the uterine fundus projected to the end of the treatment course. At the time of CT simulation, the distances to the patient's umbilicus and pubic symphysis were measured from a fixed radio‐opaque marker placed on her chin. Palpation of the uterine fundus was not achieved in our department; instead, a brief consultation with diagnostic radiology immediately prior to her CT simulation measured her uterine fundus to be 4 cm inferior to her umbilicus using a portable ultrasound unit. The total distance from each point of interest to the target volume was later determined by measuring the distance between the radio‐opaque marker and the segmented target volume in the simulation CT image. Finally, we assumed superior progression of the patient's uterine fundus at a rate of 1 cm/week and that the patient would be treated during gestational weeks 19–25.^1^ Table 1 shows the distances from the target volumes to the points of interest.

**Table 1:** Distances from field edges to points of interest for the pregnant patient reported in this manuscript

Point	Distance from inferior edge of full (50.4 Gy) treatment volume	Distance from inferior edge of boost (9 Gy) treatment volume
Pubic symphysis	76 cm	78 cm
Fundus on simulation date	59.5 cm	61.5 cm
Umbilicus	55.5 cm	57.5 cm
Fundus at end‐of‐treatment	52.2 cm	53.2 cm

Our institution's Health Insurance Portability and Accountability Act (HIPAA) Privacy Officer has reviewed this manuscript to ensure compliance with our institution's standards for protected health information. Institutional Review Board review was not required.

### 2.B Treatment plans

In an attempt to use available department resources to reduce fetal dose, four potential treatment plans were developed for the patient: (a) a 3D Conformal plan using the flattened 6 MV photon beam from a Varian TrueBeam linac (Varian Medical Systems, Palo Alto, CA, USA), including Enhanced Dynamic Wedge (EDW) wedged fields, (b) a limited‐aperture SnS IMRT plan using the flattened 6 MV photon beam from a Varian TrueBeam linac, (c) a limited‐aperture SnS IMRT plan using the flattening filter‐free 6 MV (6 MV‐FFF) photon beam from a Varian TrueBeam linac, and (d) an Accuray TomoTherapy HDA Helical IMRT treatment plan using that machine's unflattened 6 MV photon beam (Accuray, Sunnyvale, CA, USA). All treatment plans used multileaf collimator (MLC) field shaping. Treatment plans for the Varian TrueBeam were developed using Pinnacle (version 9.8, Philips Radiation Oncology Systems, Fitchburg, WI, USA) and the treatment plan for the TomoTherapy HDA system was developed using Accuray Planning Station (version 5.1.1.6, Accuray, Sunnyvale, CA, USA).

All treatment plans used specific planning techniques to reduce peripheral dose. The TrueBeam treatment plans used collimator rotations of 90° to place the distal x jaws in the patient superior–inferior direction and avoided the use of physical wedges; EDW fields may increase peripheral dose by 10%–20% in very close proximity to the treatment field, compared to 200%–400% increases for physical wedges.^4^, ^5^, ^7^, ^8^ Tertiary MLC collimation was used for all TrueBeam treatment plans. The TrueBeam plans had an isocenter placed as far cranially as possible to maximize the separation between the treatment head and the fetus^9^; additionally, couch kicks were avoided to maximize separation between the treatment head and the fetus and to avoid beam divergence toward the fetus. Beam energy was limited to 6 MV to reduce scatter, head leakage, and neutron contamination, and the flattening filter‐free beam was investigated to assess head leakage reductions from the removal of the flattening filter from the beamline.^5^, ^10^ The TrueBeam plans used minimal monitor units (MU) by avoiding highly modulated IMRT or volumetric modulated arc therapy (VMAT), minimizing the number of fields, and using SnS rather than sliding‐window technique for IMRT beams. All TrueBeam plans used six static‐gantry treatment beams. The maximum number of allowed apertures per beam for the SnS plans was four for the 6 MV beams and five for the 6 MV‐FFF beams. For TomoTherapy treatment plans, the interplay between jaw width, pitch, and modulation factor makes it difficult to generalize planning strategies to reduce peripheral dose. We pursued a minimum‐MU helical IMRT plan by using the 2.5 cm dynamic jaw width, setting the pitch to the maximum planning value of 0.43, and setting a final modulation factor of 1.4 (here, modulation factor is the ratio between maximum binary MLC leaf open time and average leaf open time). Reducing modulation factor has been shown to moderately reduce peripheral dose.^14^

### 2.C Estimates of fetal dose

**Figure 2:**
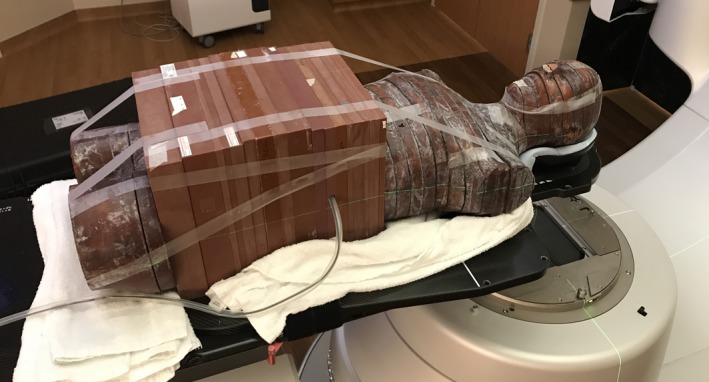
Modified anthropomorphic phantom used to measure peripheral dose for the candidate treatment plans. Photo shows placement of the ionization chamber approximately at the level of the patient's umbilicus.

Fetal dose was estimated using the prescribed doses given previously, the distances from treatment volumes to points of interest (Table 1), and properties of the treatment plans. For the TrueBeam treatment plans, Fig. 2 from Mutic and Klein and table 4 from Owrangi et al. were used to estimate peripheral dose for MLC‐shaped fields with 90‐degree collimator rotation.^4^, ^5^ The effective square field size was 11 cm for the primary treatment plans and 8 cm for the boost treatment plans. Similar data for peripheral dose distributions are also available from other sources.^1^, ^6^, ^11^, ^12^ For TomoTherapy peripheral dose estimates, perhaps the best available reference is the 2013 paper by Lissner et al.; fig. 4 from that manuscript enables estimates of peripheral dose for a variety of situations.^14^ We used fig. 4(d) from Lissner et al. to estimate peripheral dose for the patient's 18 Gy‐liter treatment volume.

### 2.D Measurement of fetal dose

Fetal dose was measured at each of the four points of interest identified in Table 1 using TLD‐100 thermoluminescent dosimeter chips from the University of Wisconsin Accredited Dosimetry Calibration Laboratory.^15^ At each point of interest, three TLD chips were placed under a 3 cm cap of bolus material on the surface of a modified anthropomorphic phantom; the phantom (prior to TLD placement) is shown in Fig. 2. The measurements from the three TLDs were averaged together for each point of interest for each treatment plan. While out‐of‐field dose has minimal dependence on depth, the superficial dose is markedly higher than the rest of the depth–dose curve; the bolus was used to position the TLDs beyond this superficial region.^11^ In addition to the TLDs, a Farmer‐type ionization chamber placed in the center of the 30 cm slabs of phantom material (depth of 15 cm) at the longitudinal level of the patient's umbilicus integrated charge over the course of each irradiation. The chamber and electrometer were carefully monitored for leakage during each measurement. After aligning the phantom to the patient's plan using 3D image guidance, each treatment plan was completely delivered (i.e., 28 fractions of primary plan plus five fractions of boost plan) to the phantom and measurement devices. The TomoTherapy deliveries also included a megavoltage CT (MVCT) scan prior to the treatment of each fraction, as would be clinically standard.

### 2.E Patient imaging

The patient was imaged for treatment planning using our department's CT simulator. A 0.5 mm lead‐equivalent apron was placed around the patient's abdomen and pelvis during her CT scan; this type of apron may reduce the CT dose to the fetus by 90% or more.^16^ Fetal dose from CT scans is exceptionally low given the collimation used on modern CT scanners and can be maintained as low as possible by choosing an appropriate imaging technique and limiting the scan range to only the necessary anatomy. Tools like the ImPACT CT Dosimetry worksheet (http://www.impactscan.org/, accessed October 5, 2017) may be valuable to estimate CT doses for a variety of organs on a standardized phantom geometry, but this tool does not provide patient‐specific dose estimates. If the scan had gone through the patient's uterus, other references can be used to estimate fetal dose.^17^ As is standard at our institution for pregnant patients, iodinated contrast was not used for the patient's simulation CT scan. Iodine‐based contrast does present some fetal risks.^18^

For image guidance during patient treatment, the intended strategy depends on the treatment. For the TrueBeam plans discussed above, orthogonal planar 2D kV‐kV imaging would be entirely sufficient to align the cranium with five of the available six degrees‐of‐freedom. If cone‐beam CT (CBCT) were required, it could be performed with minimal fetal dose on a modern TrueBeam system. Scaling organ doses previously published for a prior generation of Varian CBCT system by the CTDI ratios between the older On‐Board Imager (OBI) CBCT system and the TrueBeam CBCT system, one could estimate up to 0.0006 cGy fetal dose per “head” CBCT and up to 0.005 cGy fetal dose per “thorax” CBCT (using kidneys as a conservative surrogate for the fetus).^19^, ^20^, ^21^ TomoTherapy image guidance would be performed with the “coarse” MVCT protocol with 3 mm slice thickness to minimize the MU required for MVCT imaging.

## 3 RESULTS

The patient started treatment during gestational week 19 and finished treatment during gestational week 25, as anticipated. All four treatment plans easily met the planning goals listed in the Patient Details subsection above and were considered equivalent in terms of plan quality. Estimated fetal dose derived using the techniques described above are shown in Table 2, along with measured doses from TLDs and the ionization chamber. The estimated doses from Mutic and Klein and Owrangi et al. for the TrueBeam plans were each increased by the ratio of MU‐to‐cGy for plans 1–3 (an effective modulation factor, given our linear accelerator calibrations of 1 cGy/MU at depth of maximum dose) because published peripheral dose distributions are often reported for open, unmodulated fields. For treatment plan 1, the addition of EDW wedges made the modulation factor 1.36 (245 MU/fraction for the primary plan, 250 MU/fraction for the boost plan); for plan 2, the factor was 1.42 (255 MU/fraction for the primary plan, 261 MU/fraction for the boost plan); and for plan 3, the factor was 1.72 (313 MU/fraction for the primary plan, 290 MU/fraction for the boost plan). The estimated doses for plan 3 are not specific to the 6 MV‐FFF beam energy, as there are no published peripheral dose distributions for that beam; rather, the estimates for plan 3 are strictly applicable to the 6 MV beam energy only. Finally, the dose estimates from Owrangi et al. and Lissner et al. were corrected using the inverse‐square law to scale between the most distal point from the reference (40 cm from Owrangi and 33.4 cm from Lissner) and the point of interest for this study. Inverse‐square scaling may be appropriate given that the preponderance of peripheral dose for this study originates as leakage from the treatment head itself.^22^

**Table 2:** Estimated and measured fetal doses to the points of interest in Table 1 from the four candidate treatment plans. Fetal dose estimates were taken from Mutic and Klein,^4^ Owrangi et al.,^5^ and Lissner et al.^14^ The Lissner and Owrangi dose estimates were inverse‐square corrected, and the Mutic/Owrangi estimates were increased by the ratio of MU‐to‐cGy for plans 1–3. Estimated uncertainty was 5% for TLD measurements and 7% for ion chamber measurements

Treatment plan/point	Estimated dose	Measured dose
Plan 1: 3DC 6 MV TrueBeam		
Pubic symphysis	Mutic: 0.61 cGy Owrangi: 0.45 cGy	TLD: 0.90 cGy
Fundus on simulation date	Mutic: 0.72 cGy Owrangi: 0.72 cGy	TLD: 1.6 cGy
Umbilicus	Mutic: 0.72 cGy Owrangi: 0.83 cGy	TLD: 1.6 cGy Ion chamber: 0.79 cGy
Fundus at end‐of‐treatment	Mutic: 0.79 cGy Owrangi: 0.95 cGy	TLD: 1.6 cGy
Plan 2: IMRT 6 MV TrueBeam		
Pubic symphysis	Mutic: 0.64 cGy Owrangi: 0.47 cGy	TLD: 1.0 cGy
Fundus on simulation date	Mutic: 0.75 cGy Owrangi: 0.75 cGy	TLD: 1.6 cGy
Umbilicus	Mutic: 0.75 cGy Owrangi: 0.87 cGy	TLD: 1.5 cGy Ion chamber: 0.75 cGy
Fundus at end‐of‐treatment	Mutic: 0.82 cGy Owrangi: 0.99 cGy	TLD: 1.5 cGy
Plan 3: IMRT 6 MV‐FFF TrueBeam		
Pubic symphysis	Mutic: 0.77 cGy Owrangi: 0.57 cGy	TLD: 0.60 cGy
Fundus on simulation date	Mutic: 0.91 cGy Owrangi: 0.91 cGy	TLD: 0.80 cGy
Umbilicus	Mutic: 0.91 cGy Owrangi: 1.05 cGy	TLD: 0.90 cGy Ion chamber: 0.53 cGy
Fundus at end‐of‐treatment	Mutic: 1.00 cGy Owrangi: 1.20 cGy	TLD: 1.0 cGy
Plan 4: TomoTherapy		
Pubic symphysis	Lissner: 2.3 cGy	TLD: 1.9 cGy
Fundus on simulation date	Lissner: 3.8 cGy	TLD: 2.1 cGy
Umbilicus	Lissner: 4.3 cGy	TLD: 2.3 cGy Ion chamber: 1.6 cGy
Fundus at end‐of‐treatment	Lissner: 4.9 cGy	TLD: 2.6 cGy

The measured doses for the four plans at the four points of interest are also given in Table 2. The measured doses are in fairly good agreement with the estimated doses (adjusted for plan modulation and inverse‐square law where necessary). The uncertainty in the TLD measurements was estimated to be 5%, given the very low doses used to calibrate the TLD batch. The ion chamber leakage signal was estimated to be approximately 6.5% of the measurement signal; although this leakage was nulled at the electrometer, we estimate the total uncertainty in our ion chamber measurements to be approximately 7%, including leakage. In general, the umbilicus‐level TLDs reported higher doses than the ion chamber at the same longitudinal position. The TrueBeam plan measurements included the full plan delivery (28 fractions primary plus five fractions boost), while the TomoTherapy plan measurements included the full plan delivery plus 33 clinically appropriate MVCT scans. The maximum reported doses for the four plans were 1.6 cGy for plans 1 and 2, 1.0 cGy for plan 3, and 2.6 cGy for plan 4. Based on these measurements, treatment plan 3 (6 MV‐FFF TrueBeam SnS IMRT) was selected for patient treatment. Given the very low fetal doses, no external fetal shielding was used for patient treatment.

Based on the gestational age of the fetus at the time of irradiation and the low fetal dose levels, minimal effects are expected for the fetus.^1^, ^2^ The dose levels are below threshold doses for malformation (including microcephaly) and drop in intelligence quotient. Based largely on follow‐up observation of the atomic bomb survivor cohort, the absolute risk of inducing a fatal childhood cancer before the age of 19 may be as high as 0.06% per 1 cGy.^2^ Table 4 from ICRP Report 84 shows that the risk of a fatal childhood cancer from the 1 cGy fetal dose for treatment plan 3 in this study increases the 0.3% baseline risk to 0.4%. The patient was consented for treatment only after this information was provided to her and her questions were answered satisfactorily by the attending physicist and physician. All four treatment plans investigated herein produced maximum measured fetal doses less than the 5 cGy limit given in Task Group Report 36 table VI for “little risk of damage” to the fetus, and all four plans were clinically acceptable to the physician.^1^

## 4 DISCUSSION

This study reports on a brain tumor patient treated recently in our clinic to a total dose of 59.4 Gy in 33 fractions during her second trimester of pregnancy. Four candidate treatment plans were created using a variety of planning strategies intended to reduce fetal dose. Estimates of fetal dose for each plan were made using published literature, and measurements of fetal dose were made for each plan using a modified anthropomorphic phantom. Measurements and estimates were performed at points of interest that bracketed the potential locations of the fetus during the duration of the patient's treatment. The treatment plan with the lowest measured fetal dose was selected for patient treatment.

The estimated doses to the points of interest were adjusted using the MU‐to‐cGy ratio for the TrueBeam treatment plans, as well as using the inverse‐square law when peripheral dose estimates were not available at the distances given in Table 1. Both of these corrections are appropriate under the assumptions that nearly all the fetal dose in this case originates from the treatment head as leakage and that leakage is directly proportional to MU. Even after these corrections, though, several shortcomings remain in the estimation of peripheral doses. First, the documents used to estimate peripheral dose for this patient relied on measurements from a prior generation of Varian delivery systems where head shielding was constructed differently than in a modern TrueBeam system. Second, there is no published guidance on the distribution of peripheral dose from the 6 MV‐FFF beam energy in a TrueBeam delivery system, but the removal of the flattening filter from the beam line will certainly reduce direct head leakage. This was observed in the plan measurements performed by Owrangi et al., where changing from 6 MV to 6 MV‐FFF for a 3D brain plan resulted in a 20% reduction in unshielded fetal dose.^5^ As a note of caution, using 6 MV‐FFF for targets of any reasonable size will require more plan modulation (and therefore more MU) to achieve a uniform dose distribution than a 6 MV plan: the nonuniform profile of the 6 MV‐FFF beam must be “modulated out.” Finally, the peripheral dose estimates for the TomoTherapy plan relied on data for a 30 Gy‐liter plan, where our patient's treatment plan was 18 Gy‐liter. This certainly explains some of the discrepancy between TomoTherapy estimated and measured doses.

The measured doses from the TLDs in this study are markedly higher than the corresponding umbilicus‐level ionization chamber doses for the same plans. This is likely a result of the relatively small pieces of bolus placed over the TLDs. Although single static‐field peripheral dose distributions have little depth dependence after the depth of maximum dose, it is possible that composite plan peripheral dose is distributed differently. In any case, the TLDs provide a conservative estimate of fetal dose, should the fetus be positioned close to the patient's anterior surface.

No fetal radiation shield was used for patient treatment; when Owrangi et al. measured fetal doses from example plans with and without a unique and presumably expensive fetal shield, fetal dose was reduced by around 30% at most for brain plans.^5^ This limited reduction is presumably due to the contribution of primary beam‐quality head leakage to fetal dose for this body. The unshielded fetal dose reported by Owrangi et al. was 0.81 cGy for a 6 MV 3D plan, 0.65 cGy for a 6 MV‐FFF 3D plan, 3.2 cGy for an IMRT plan, and 2.3 cGy for a VMAT plan (all assessed at a point 50 cm from the center of the PTV).^23^

Other authors have reported fetal dose estimates in the literature for a variety of treatment sites, including brain. Sharma et al. report on the treatment of a 30‐year‐old patient with a nonfunctioning pituitary adenoma; planning details are not fully apparent, but the fetal dose was measured to be 2 cGy from the 45 Gy treatment course.^24^ Horowitz et al. reported in 2014 on a 37‐year‐old patient being treated for grade 4 glioblastoma.^25^ Postoperative radiation delivered 60 Gy in 30 fractions using IMRT with three co‐planar fields on a Varian Clinac; fetal dose was measured (both with and without shielding) to be 2 cGy over the course of treatment. For head and neck treatments, Owrangi et al. provided several treatment plan options, including a 6 MV VMAT plan delivering 66 Gy in 33 fractions; the fetal dose was 11 cGy to a point 40 cm from isocenter.^5^ Ramsey et al. published a TomoTherapy head and neck phantom study for a TomoTherapy Hi‐ART system; helical IMRT was used with a 2.5 cm jaw setting, a pitch of 0.333, and a modulation factor of 2.5.^26^ For a 66 Gy course, the Ramsey study would have calculated 12.5 cGy to the fetus. Josipović et al. reported in 2009 on a 33‐year‐old patient, 27 weeks pregnant, with advanced head and neck cancer.^9^ The treatment dose was 68 Gy in 34 fractions using IMRT with 4 MV photons from a Varian Clinac 2300; the fetal dose was estimated to be between 9 and 14 cGy using a very large metal block cantilevered from the face of the treatment system as shielding. Numerous reports also exist for fetal dose from radiation therapy for breast cancer, showing fetal doses of 1.0 cGy (for shielded electron treatment) up through 8.5 cGy (for photon IMRT).^27^, ^28^, ^29^

## 5 CONCLUSIONS

We recently treated a pregnant patient for a resected grade 3 astrocytoma during gestational weeks 19–25. During the treatment planning process, we developed four potential treatment plans including plans for a Varian TrueBeam system and an Accuray TomoTherapy HDA system. Treatment planning techniques to minimize peripheral dose were used for all four plans. Fetal doses were estimated using available literature and measured using TLDs and an ionization chamber in a modified anthropomorphic phantom. Across the four plans, at relevant points of interest, fetal doses ranged from 0.6 to 2.6 cGy; the fetal doses from each of the treatment plans were deemed acceptable. The fetal dose over the course of treatment was no more than 1.0 cGy with the selected treatment plan (6 MV‐FFF SnS IMRT on a Varian TrueBeam). Relevant treatment planning strategies and treatment delivery considerations were discussed with regard to the management of pregnant patients.

## ACKNOWLEDGMENTS

The authors acknowledge Dr. Wesley Culberson and Clifford Hammer in the UW Accredited Dosimetry Calibration Laboratory for their helpful discussions on the use of TLDs to measure low doses.

## CONFLICT OF INTEREST

The authors report no conflicts of interest.
